# UAP56 associates with DRM2 and is localized to chromatin in Arabidopsis

**DOI:** 10.1002/2211-5463.12627

**Published:** 2019-04-05

**Authors:** Jacinthe Azevedo, Claire Picart, Laurent Dureau, Dominique Pontier, Sylvie Jaquinod‐Kieffer, Mohamed‐Ali Hakimi, Thierry Lagrange

**Affiliations:** ^1^ LGDP‐UMR5096 CNRS Perpignan France; ^2^ LGDP‐UMR5096 Université de Perpignan Via Domitia France; ^3^ Laboratoire Biologie Grande Echelle Institut de Biosciences et Biotechnologies de Grenoble UMR_S 1038 CEA, INSERM Université Grenoble Alpes France; ^4^ Institute for Advanced Biosciences (IAB) Team Host‐pathogen Interactions and Immunity to Infection INSERM U1209 CNRS UMR5309 Université Grenoble Alpes France

**Keywords:** Arabidopsis, chromatin, DNA methylation, DRM2, RdDM, UAP56

## Abstract

Repeated sequence expression and transposable element mobilization are tightly controlled by multilayer processes, which include DNA 5′‐cytosine methylation. The RNA‐directed DNA methylation (RdDM) pathway, which uses siRNAs to guide sequence‐specific directed DNA methylation, emerged specifically in plants. RdDM ensures DNA methylation maintenance on asymmetric CHH sites and specifically initiates *de novo* methylation in all cytosine sequence contexts through the action of DRM DNA methyltransferases, of which DRM2 is the most prominent. The RdDM pathway has been well described, but how DRM2 is recruited onto DNA targets and associates with other RdDM factors remains unknown. To address these questions, we developed biochemical approaches to allow the identification of factors that may escape genetic screens, such as proteins encoded by multigenic families. Through both conventional and affinity purification of DRM2, we identified DEAD box RNA helicases U2AF56 Associated Protein 56 (UAP56a/b), which are widespread among eukaryotes, as new DRM2 partners. We have shown that, similar to DRM2 and other RdDM actors, UAP56 has chromatin‐associated protein properties. We confirmed this association both *in vitro* and *in vivo* in reproductive tissues. In addition, our experiments also suggest that UAP56 may exhibit differential distribution in cells depending on plant organ. While originally identified for its role in splicing, our study suggests that UAP56 may also have other roles, and our findings allow us to initiate discussion about its potential role in the RdDM pathway.

**Abbreviations**
GST: glutathione‐*S*‐transferase
HRP: horseradish peroxidase
IP: immunoprecipitation
lncRNA: long non‐coding RNA
MNase: micrococcal nuclease
RdDM: RNA‐directed DNA methylation
UAP56a/b: U2AF56 Associated Protein 56

In plants, DNA methylation is maintained by a sophisticated network involving the cooperation of several specialized DNA methyltransferases targeting cytosines in specific sequence contexts 1. Three distinct families of DNA methyltransferases cooperate to ensure cytosine methylation maintenance in plants. The first one is MET1, which is a conserved DNA methyltransferase homologous to mammalian DNMT1, which targets cytosines in a CG context 2. Unlike their animal counterparts, plants also target methylation in a non‐CG context. Thus, the chromomethylases proteins, mainly CMT2 and CMT3, bind histone H3K9 dimethylation marks and methylate non‐CG cytosines. CMT3 shows a preference for cytosines in a CHG context (where H is any base but G), whereas CMT2 carries out essentially DNA methylation on CHH sequences especially enriched in pericentromeric regions 3, 4, 5. In addition, one specific pathway has emerged specifically in the plant kingdom, known as RNA‐directed DNA methylation (RdDM) 6. The RdDM pathway contributes to the maintenance of methylation in a CHH context, targeting preferentially small transposable elements located all along the chromosome arms through the action of DRM2, a DNA methylation enzyme that is orthologous to mammalian DNMT3. Interestingly, DRM2 is the only enzyme that can establish *de novo* methylation in all sequence contexts on a naive DNA copy. The RdDM pathway is considered as an archetypal pathway for RNA‐mediated chromatin silencing and refers to a process in which repeat‐derived 24‐nt siRNAs guide DNA methylation, histone modifications and gene silencing to transposable elements. It relies on the action of two plant‐specific RNA polymerase II (Pol II)‐related enzymes known as Pol IV and Pol V. These specialized RNA polymerases exhibit an affinity for peculiar epigenetic signatures or elements associated to heterochromatic regions 7, 8, 9, 10, 11, 12, thereby maintaining targets in a silent state. Pol IV initiates the production of 24‐nt‐long siRNAs, which once loaded into their cognate AGO protein, guide DNA methylation to homologous Pol V transcribed loci. Pol V acts downstream of the effector phase, and as a reinforcement loop to Pol IV's action 13. The spatiotemporal coordination of the effector phase is still under discussion, the first question being the recruitment step of the AGO–siRNA complex to RdDM targets, a prerequisite for triggering DNA methylation. The long‐standing model suggests contributions of both protein–protein and RNA–siRNA interactions. Thus, Pol V non‐coding transcript is predicted to act as a scaffold to guide AGO4–siRNA in the vicinity of the RdDM targeted loci 14, 15. The SiRNA–AGO4 complex is also caught by WG/GW repeat motifs, called the Ago hook, present in the large Pol V subunit (NRPE1) and in the SPT5L elongation factor 16, 17, 18. However, a revisited model has been recently proposed, showing that AGO4–siRNA may access DNA directly via GW/WG protein interactions 19. Although these observations are not mutually exclusive, the nature of siRNA base‐pairing may condition the characteristics of some accessory proteins impacting subsequent steps including DNA methylation. This point raises questions about the recruitment as well as the modus operandi of the *de novo* DNA methyltransferase DRM2, two key steps that remain poorly investigated so far. Two factors are known to associate and to cooperate with DRM2. An evident connection has been established between DRM2 and AGO4 20. A co‐transcriptional slicing activity has been assigned recently to AGO4 21 and this activity challenges the importance of a stable RNA–siRNA tethering in DRM2 recruitment in the vicinity 22. This is in some ways difficult to reconcile with a previous scenario proposing a sequential recruitment of AGO4 and the RNA binding protein IDN2 to Pol V transcripts prior to DRM2 23. The second identified DRM2 partner is RDM1, a single strand DNA methyl binding protein which presents the singularity of acting at both early and late stages of the RdDM effector phase. This factor has also been involved in the production of Pol V‐ and Pol II‐dependent scaffold transcripts of RdDM target loci, Pol II acting mainly on alternative RdDM targets through non‐canonical pathways 24, 25. Pol II and Pol V targets show also different organizations or compartmentalizations into nucleus 26, 27.

To uncover new factors acting on this late effector phase, we focused our investigation on DRM2. We set up two DRM2 biochemical purification strategies to bypass genetic screen limitations such as redundancy. Among the candidates isolated from both approaches, we identified highly conserved DEAD box RNA helicases, known to impact the splicing and the export of Pol II‐dependent transcripts, U2AF56 Associated Protein 56 (UAP56a/b). These proteins are encoded by two tandemly duplicated genes in Arabidopsis, *UAP56a* and *UAP56b*. Subcellular localization assays and chromatin isolation techniques confirmed that the nuclear UAP56 fraction and DRM2 share the same purification features, supporting our *in vitro* and *in vivo* interaction assays. Finally, all our attempts to get a *uap56* double KO mutant failed, raising the question of the viability of such plants, as has been observed in yeast and animal counterparts, thereby limiting *a fortiori* the genetic analysis of this partnership and its relevance *in vivo*.

## Material and methods

### Cloning and plant methods

All *Arabidopsis thaliana* mutant lines used in this study are in the Columbia ecotype background. Plants were either grown in soil or cultivated *in vitro* on ½ MS medium plus agar (Duchefa), supplemented with hygromycin (25 μg·L^−1^) for transgenic *pDRM2‐DRM2‐FLAG‐HA*/*drm1drm2* plant selection. For *in vitro* culture, seeds were stratified for 48 h at 4 °C before incubation at 20 °C with a 16 h light–8 h dark cycle (130 μE·m^−2^·s^−1^ light, LEDs with white 4500 K spectrum, from Vegeled). Arabidopsis and *Nicotiana benthamiana* plants were grown on soil at 20 °C with a 16 h light–8 h dark cycle (100 μE·m^−2^·s^−1^ light, fluorescent bulbs with white 6500 K spectrum, from Sylvania) and 60–75% humidity. Two independent knockout mutant lines were used for each UAP56 gene during this work (GABI_528B02 and WiscDsLox413‐416C15 for *UAP56a*; Sail883C11 and GABI_110E12 for *UAP56b*). The list of primers used for genotyping is presented in Table S2.

All coding or genomic sequences cloned for this work were amplified using primers listed in Table S2 with the Phusion enzyme (New England Biolabs) on Arabidopsis Col‐0 cDNA or genomic DNA templates and sequenced. The full‐length *DRM2* genomic amplicon was introduced into a XmaI–BamHII‐digested modified FLAG‐HA vector (pCAMBIA 1300 backbone). The construct was then used to transform the *drm1drm2* mutant by the floral dip method.

*DRM2* and *UAP56a* cDNA were inserted into SalI–PstI‐digested binary vector derived from a pCAMBIA1300, generating C‐ter fusion with RFP or GFP under control of the constitutive promoter *p35S*. These clones were introduced into *Agrobacterium tumefaciens* strain GV3101, and suspensions prepared in 10 mm MES, 10 mm MgCl_2_ with an absorbance of 0.8 were used to infiltrate *N. benthamiana* leaves. The coding sequence of *UAP56a* was also inserted into a EcoRI–HindIII‐digested pET‐28a(+) and pET‐41a(+) (Novagen) to produce recombinant proteins in *Escherichia coli* BL21 strain.

CRISPR constructs were designed using a sgRNA which targets *UAP56a* and *UAP56b* first exon (Table S2). The CRISPR/CAS9 system used in our work was adapted from Zhang *et al*., 2016 28 with a *AtU6* promoter upstream sgRNA module, and a double *p35S* promoter to drive *CAS9* expression. The T1 population was first screened on ½ MS plates supplemented with kanamycin (50 mg·L^−1^) for CAS9 cassette selection. Resistant seedlings were then transplanted to soil prior to genomic DNA extraction. In parallel, CAS9 protein was detected by immunoblotting using anti‐Flag‐horseradish peroxidase (HRP) antibody (Sigma‐Aldrich). Mutations generated by CAS9 nuclease were analyzed on 60 T1‐resistant plants by sequencing PCR products surrounding the sgRNA target site (Table S2). Among them, only one T1 plant presented a mutation at the predicted site, confirmed by two independent sequencing analyses (insertion of an A base between the third and the fourth base following the PAM sequence). After self‐fertilization, 84 T2 plants were analyzed (with and without kanamycin selection) following the same procedure.

### Complementation analyses

All complementation analyses were performed on flowers.

Total RNA was extracted using Trizol according to the manufacturer's instructions (MRCgene), and low molecular mass northern blots were carried out as described in Bies‐Etheve *et al*. 17. The probes used to detect *U6* and *5S* siRNA are presented in Table S2.

DNA methylation analyses by CHOP‐PCR are detailed in Lahmy *et al*. 19, but starting digestion with 200 ng of genomic DNA (DNeasy Plant mini Kit; Qiagen). Primers used to test RdDM target amplification after HaeIII digestion are also listed in Table S2, and AT2G19920 was designed as an undigested control.

Protein samples from wild‐type and transgenic lines were extracted according to the method of Hurkman and Tanaka 29, quantified and subjected to immunoblot analysis using antibodies raised against peptides described in the following section.

### Antibodies

All custom made antibodies were prepared in rabbits by Eurogentec (Eurogentec SA). Rabbit antisera were produced against DRM2 peptides EP112214 (NSDDEKDPNSNENGS) and EP112215 (ESKGEPRSSVDDEPI) following their double‐X immunization program and then affinity‐purified on EP112215. Antibodies for NRPD1 detection were also raised in rabbits against EP112201 (ESKGEPRSSVDDEPI) peptide and affinity purified by Eurogentec. His‐tagged UAP56 protein was produced from pET‐28a‐*UAP56* in BL21 *E. coli* strain and purified with His‐bind resin following the supplier's instructions (Millipore). Anti‐UAP56 serum was then produced in rabbits using this recombinant protein as antigen. Anti‐AGO4 antibodies were previously used by Lahmy *et al*. 19. Monoclonal antibody 8WG16 (ab817; Abcam) was used to detect NRPB1; histone H3 (ab1791; Abcam) and UGPase polyclonal antibodies (AS05 086, Agrisera) were also used for nucleus and cytoplasm controls. Affinity‐purified anti‐HA antibodies coupled to HRP (Sigma‐Aldrich, clone HA‐7) were used to detect DRM2 in transgenic tagged lines.

### Protein analysis and detection

Protein quantification was performed using the Bradford assay according to the supplier's instructions (Bio‐Rad).

Eluates from immunoprecipitation or columns were denatured with Laemmli denaturing buffer and separated by SDS/PAGE using pre‐cast gradient gels with MOPS/SDS running buffer (NuPAGE® Novex®4–12% Bis‐Tris polyacrylamide gel, Invitrogen, Thermo Fisher Scientific). Proteins were either stained on gel using ProteoSilver Plus silver stain Kit (Sigma‐Aldrich), or electrotransferred onto a poly(vinylidene difluoride) membrane (Immobilon‐P; EMD Millipore) and immunodetected by colorimetry (alkaline phosphatase conjugated goat secondary antibodies from Promega, and NBT‐BCIP from Amresco as substrate). For identification by mass spectrometry (MS), proteins were separated by SDS/PAGE (NuPAGE® Novex®4–12% Bis/Tris polyacrylamide gel) in a MES/SDS running buffer (Invitrogen), and stained with Colloidal Blue Staining Kit (Invitrogen).

### Chromatographic purification and protein analysis methods

For the FLAG immunoprecipitation (IP) strategy, *pDRM2‐DRM2‐FLAG‐HA*/*drm1drm2* flower whole cell extracts (2.5 g) were prepared in BC500 buffer + 0.1% NP‐40 (20 mm Tris/HCl, pH 8.0, 500 mm KCl, 10% glycerol, 1 mm EDTA, 1 mm DTT, 0.5 mm PMSF, MG132 10 μm and EDTA‐free proteases inhibitor cocktail from Roche), and then incubated with anti‐FLAG M2 affinity gel (0.5 mL; Sigma‐Aldrich) for 1.5 h at 4 °C. Beads were washed with 15 column volumes (cv) of BC500 buffer followed by 15 cv of PBS. Bound peptides were eluted stepwise with 250 μg·mL^−1^ 3×FLAG peptide (Sigma‐Aldrich) diluted in BC100 buffer. Flag IP is controlled with silver nitrate gel staining and western blotting. Eluted fractions were then pooled and precipitated using TCA (10% final concentration; Sigma‐Aldrich), and the pellet washed with cold acetone.

A conventional chromatographic purification strategy was performed starting from Col‐0 flower whole cell extracts obtained from 20 g of frozen material prepared in BC100 buffer. All through this process, protein separation was visualized on a gel by silver staining, and DRM2 protein was detected by immunoblotting with anti‐DRM2 antibody (dilution 1/1000). First, proteins were separated through a 250 mL phosphocellulose column (P11 resin from Whatman), using step gradients of salt concentration (0.1, 0.3, 0.5, 1 m KCl). About 180 mL of 0.1 m KCl elution fraction was then loaded onto a DEAE Sephacel column (10 mL bed volume; resin from GE Healthcare) and eluted in a step gradient with 0.5 and 1 m KCl. Fractions corresponding to 0.5 m KCl elution were dialysed in BC75 buffer and then separated on a MonoQ GL5/50 column (GE Healthcare) using a salt linear gradient (0.1–0.5 m KCl on 20 cv). Fractions surrounding the DRM2 elution peak were pooled and fractionated on two successive exclusion chromatography columns (Superdex200 HR 10/30; GE Healthcare) equilibrated in BC500 buffer + 0.5% NP40. Fractions 27–29 were finally pooled and concentrated (Nanosep 10 K; PALL) before tandem mass spectrometry (MS/MS) analysis. It should be noticed that we always eluted DRM2 as a unique peak after each column.

### Mass spectrometry‐based proteomics

Protein bands were excised from colloidal blue‐stained gels and treated with DTT and iodoacetamide to alkylate the cysteines before in‐gel digestion using modified trypsin (sequencing grade; Promega). The resulting peptides from individual bands were analysed by online nanoLC‐MS/MS (UltiMate 3000 coupled to LTQ‐Orbitrap Velos Pro; Thermo Fisher Scientific) using a 25‐min gradient. Peptides and proteins were identified with Mascot and validated with irma software (v 1.31.0) through searches against an Arabidopsis database 30. Peptides whose Mascot score was greater than 18 were marked as significant and proteins identified with a single or two peptides were considered only if they had a score of 50 and 25, respectively.

### Validation interaction assays

For glutathione‐*S*‐transferase (GST) pull‐down assays, GST and GST–UAP56 proteins were produced from pET‐41a(+) backbones (Novagen) in *E. coli* BL21. Expression was induced for 3 h with 1 mm IPTG in cells grown at 37 °C until reaching an absorbance of 1. Cells were disrupted using a Vibracell sonicator (Bioblock) and GST‐fusion proteins were purified by glutathione Sepharose 4B (VWR). Sixty micrograms of purified GST or GST–UAP56 protein was immobilized onto 60 μL of glutathione Sepharose 4B, and the coated beads were washed with PBS and equilibrated with EB150 buffer (50 mm Tris/HCl pH 7.5, 150 mm NaCl, 5 mm MgCl_2_, 5% glycerol, 0.1% NP40, EDTA‐free protease inhibitor cocktail from Roche). A total of 750 μL of flower *pDRM2‐DRM2‐FLAG‐HA*/*drm1drm2* whole‐cell extract was applied to the GST–CTD beads and mixed for 3 h at 4 °C on a rotating wheel. The beads were then washed three times with IP buffer, and bound proteins eluted by competition using 10 mm reduced glutathione. All samples were separated by 10% SDS/PAGE and subjected to western blotting.

To perform anti‐UAP56 IP, 0.5–1 g of inflorescences was ground in liquid nitrogen and homogenized in the two to three volumes of EB150 buffer, supplemented with protease inhibitor cocktail EDTA free (Roche) and 10 μm MG132 (Sigma‐Aldrich). Cell debris was removed by centrifugation at 20 000 ***g*** at 4 °C for 30 min. The clarified lysate was incubated for 3 h at 4 °C in a rotating wheel at 7 r.p.m., in the presence or not of antibodies. The result of two different dilutions of anti‐UAP56 sera (1/500 or 1/1000) in the tested IPs is shown here. Dynabeads Protein G from Invitrogen (30 μL·IP^−1^) were then added and incubated for an additional 3–4 h. Beads were washed once with 1 mL of EB150 and tubes changed before elution. Immunoprecipitates were eluted with two volumes of 0.1 m glycine/HCl pH 2.5 (Sigma‐Aldrich) and IP products were neutralized with 1 m Tris prior to denaturation in Laemmli buffer for 10 min at 95 °C. Input, IPs and corresponding unbound protein fractions were separated by 10% SDS/PAGE, and subjected to western blotting. DRM2 was detected using anti‐HA‐HRP antibodies (clone HA‐7; Sigma‐Aldrich) and UAP56 serum was diluted at 1/5000. Regarding UAP56 molecular mass (about 50 kDa), trueblot anti‐Rabbit IgG‐HRP (Rockland) was used to check immunoprecipitated UAP56 proteins by western blotting, thereby avoiding cross reaction with IgG heavy chains co‐migrating into the gel.

### Confocal microscopy observation

Agroinfiltrated *N. benthamiana* leaves (as described above) were used to monitor the subcellular distribution of DRM2–RFP and UAP56–GFP fusion proteins, 48 h post‐infiltration. Observations and acquisitions were performed using an LSM700 (Zeiss) confocal microscope with the following excitation and emission wavelengths: RFP: 555 nm/560–700 nm and GFP: 488 nm/490–555 nm (band pass filter).

### Cellular and chromatin fractionation analyses

The subcellular fractionation method was adapted from Watson and Thompson 31 with the following modifications. Flowers were frozen and ground with liquid nitrogen, and then homogenized in 3–5 mL of HB·g^−1^ of material (HB: 20 mm MOPS/NaOH pH 7, 0.5 m hexylene glycol, 10 mm MgCl_2_ and 5 mm 2‐mercaptoethanol). The suspension was filtrated through four layers of Miracloth (Calbiochem) and one layer of 25 μm mesh. Triton X‐100 was then added dropwise until reaching a 0.5% final concentration, with gentle swirling. A first centrifugation (10 min, 1000 ***g***, 4 °C) sedimented a concentrated crude nuclei fraction. The supernatant corresponds to nuclei‐depleted total extract, and was used here as the cytosolic fraction. The nuclei pellet was resuspended in HB + 0.5% Triton X‐100 and purified through a 60% Percoll cushion, prepared in HB + 0.5% Triton X‐100 (centrifugation 400 ***g*** for 30 min, 4 °C). The nuclei pellet was washed in 10–12 volumes HB + 0.5% Triton X‐100, prior to a final sedimentation (10 min, 1000 ***g***, 4 °C) and resuspension in HB + 0.5% Triton X‐100. Proteins were quantified using the Bradford assay (Bio‐Rad). Inputs of nuclei used for subsequent chromatin‐related analyses were prepared according to this method.

Native chromatin extraction was performed as described in Henikoff *et al*. 32, with minor modifications as we used HB buffer supplemented with 1 mm CaCl_2_ for a micrococcal nuclease (MNase) reaction. Ten microliters of each chromatin salt‐extracted fraction was used to isolate DNA and check MNase digestion efficiency by electrophoresis on a 2% low melting agarose/TBE1X gel. The chromatin soluble fraction was extracted as previously described in Lahmy *et al*. 19.

## Results and Discussion

### Identification of DRM2‐associated proteins

**Figure 1:**
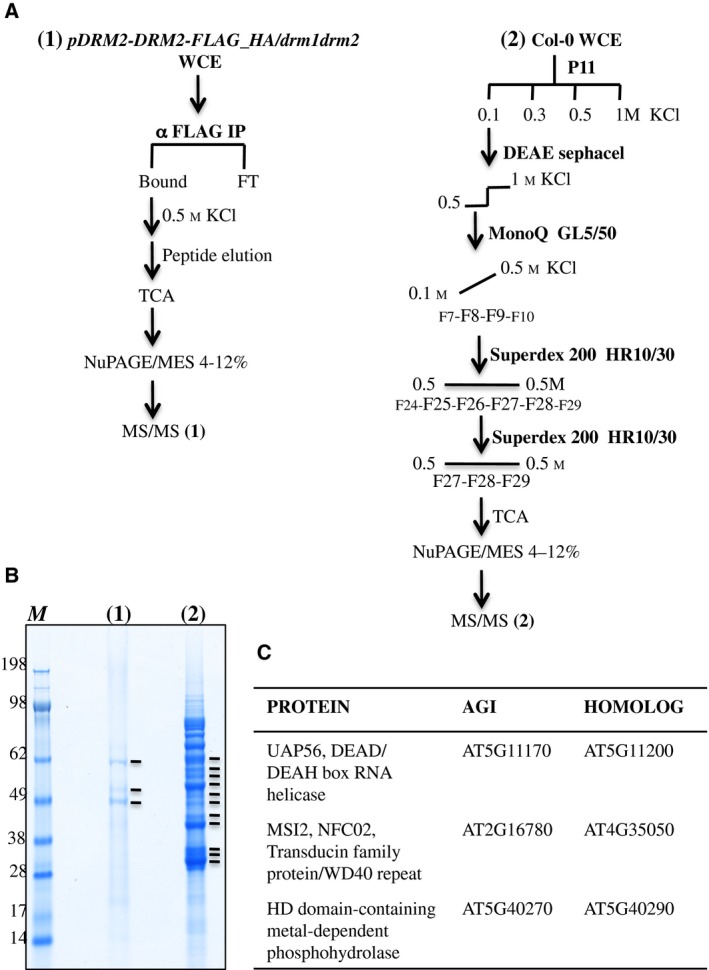
Purification of DRM2‐associated proteins from Arabidopsis* *flowers. (A) Purification schemes for both strategies used to isolate DRM2: a tag affinity purification from complementing *pDRM2‐DRM2‐FLAG‐HA* lines (1) and a fractionation by chromatography from wild‐type plants (2). Fractions selected by western blotting for subsequent column separation are indicated. Proteins finally collected at the end of both procedures were precipitated with TCA and separated by SDS/PAGE (4–12%). (B) Colloidal blue staining of proteins isolated in both methods. Bands analysed by MS/MS are indicated (14 bands). (C) List of proteins encoded by multigene families identified in common from both strategies.

To find new proteins associated with DRM2 and perhaps required for its action, we compared the results obtained from two different DRM2 purification strategies, thereby increasing confidence in the identified relevant partners. The first strategy relies on a tag affinity procedure (Fig. 1A); to do that, we generated tagged *pDRM2‐DRM2‐FLAG‐HA* lines complementing *drm1drm2* defects such as restoration of 5S‐derived siRNA accumulation and DNA methylation at RdDM target loci (Fig. S1A–C). Flag IP was carried out starting from flower protein extracts. Bound proteins were eluted by Flag peptide competition, separated by denaturing electrophoresis and stained with colloidal Coomassie. This procedure led to a confident and discrete elution pattern, allowing us to focus on three main bands for further MS/MS analysis (Fig. 1B). The second strategy followed conventional biochemistry schemes and led to isolate DRM2 and associated proteins through successive ion exchanger and exclusion chromatography columns. The DRM2 protein was monitored by immunoblotting using anti‐DRM2 antibody (Fig. S1D) in the successive fractions (Figs 1A and S2 for detailed procedure). At the end of this process, the proteins separated on a denaturing gel and stained with colloidal blue displayed a complex pattern corresponding to factors co‐enriched with DRM2, sharing the same biochemical properties (Fig. 1B). To focus on new potential partners and to be able to compare results obtained from both strategies, 11 supplemental bands were analysed by MS/MS between 62 and 30 kDa (indicated in lane 2 of Fig. 1B), covering largely the range of protein size obtained after Flag IP (lane 1, Fig. 1B). Nevertheless, this selection excluded the possibility of identifying AGO4. Five proteins co‐purify with DRM2 in both experiments (Table S1), and strikingly three out of five present homologs (Fig. 1C). Among them, we paid particular attention to UAP56, a conserved DEAD box RNA helicase, encoded by two neighboring genes, *UAP56a* and *UAP56b* (AT5G11170 and AT5G11200, respectively) producing proteins harboring 100% amino acid identity. In Arabidopsis, the association of UAP56 to RNA trafficking complexes THO/TREX is conserved 33, and it is relevant to note that some THO/TREX components have been isolated from two independent post‐transcriptional gene silencing genetic screens, showing an impact on processing of RNAs producing secondary siRNA such as transgenes, *TAS* and endogenous inverted repeats loci 33, 34. Originally identified in yeast for its role in mRNA splicing 36, 37 and export 38, UAP56 exhibits ambivalent actions in Drosophila nucleus promoting the release of mRNA from transcription sites and regulating the spread of chromatin 39.

**Figure 2:**
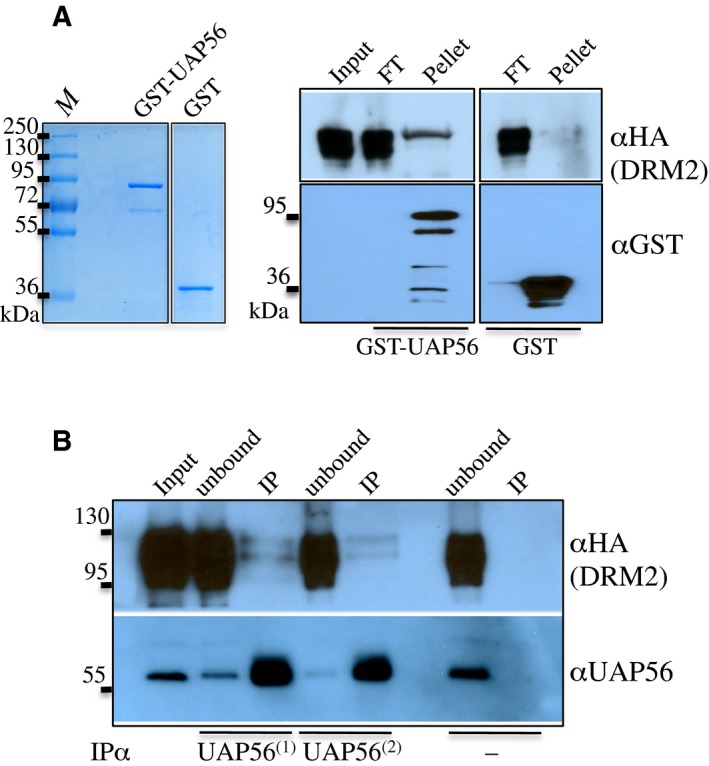
UAP56 associates with DRM2 *in vitro* and *in vivo*. (A) GST pull‐down performed in presence of *pDRM2‐DRM2‐FLAG‐HA* whole cell extract (flowers). Equimolar amounts of purified GST or GST–UAP56 were immobilized onto resin. The recombinant proteins were visualized by colloidal blue gel staining prior to pull‐down experiment and their immunodetection was performed using anti‐GST antibody. Bound proteins were detected by immunoblotting using an anti‐HA antibody. FT, flowthrough. (B) Co‐IP experiment using anti‐UAP56 antibodies applied to *pDRM2‐DRM2‐FLAG‐HA* flower whole cell extract. The same input was divided into three reactions to test two dilutions of anti‐UAP56 (1/500 and 1/1000) in presence of Protein G Dynabeads, and the third reaction without antibody was used as negative control. Each corresponding unbound and IP fraction was analysed by western blotting using anti‐HA‐HRP to detect DRM2, and anti‐UAP56.

More recently, its role was extended to post‐transcriptional silencing of transposons by the Piwi pathway in Drosophila germline, facilitating the stabilization and the export of piRNA precursors to the cytosolic nuage structure, the site of piRNA production and transposon degradation 40. These intriguing observations led us to focus our work on this DRM2 partner candidate. To validate the DRM2 and UAP56 association, we combined both *in vitro* and *in vivo* approaches, using respectively GST pull‐down assays and reverse co‐IP experiments. GST–UAP56 recombinant protein was produced from *E. coli* and purified using immobilized glutathione resin affinity. GST–UAP56 and the negative control GST were then used as bait to pull down protein extracts obtained from *pDRM2‐DRM2‐FLAG‐HA* flowers. In these conditions, UAP56 was shown to interact specifically with DRM2 *in vitro* (Fig. 2A). Reverse co‐IPs were then performed to validate this *in vitro* result. UAP56 was thereby immunoprecipitated from *pDRM2‐DRM2‐FLAG‐HA* flower protein extracts. Using previously described anti‐UAP56 serum 41, we were able to detect DRM2 in the UAP56 IP eluates (Fig. 2B). Collectively these data support a partnership between the main *de novo* DNA methyltransferase and UAP56 in Arabidopsis flowers.

### The UAP56 protein is partitioned between the nucleus and the cytoplasm in reproductive organs

**Figure 3:**
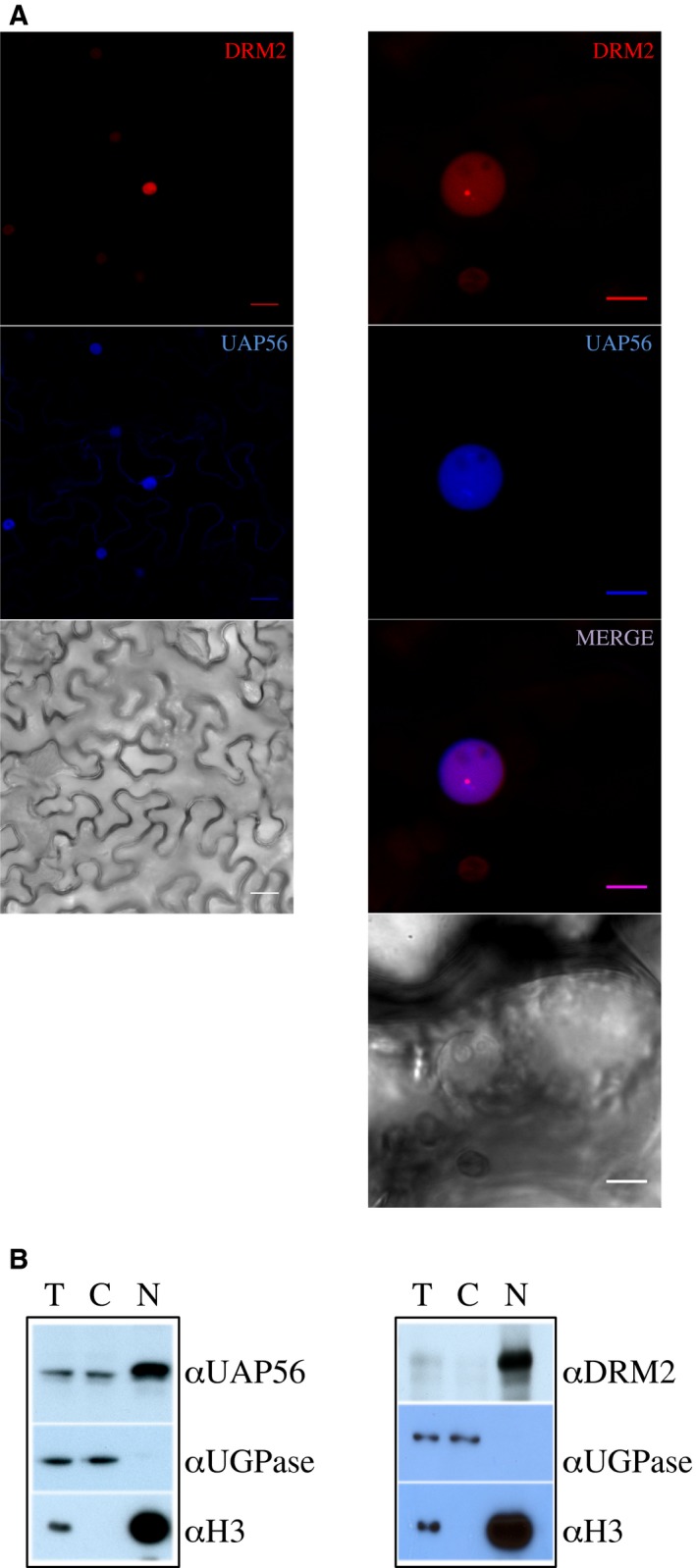
UAP56 and DRM2 proteins partially co‐localize in the nucleus. (A) Distribution in cells visualized by confocal microscopy in *N. benthamiana* leaves expressing transiently *p35S‐UAP56‐GFP* and *p35S‐DRM2‐RFP* constructs. RFP and GFP signals are depicted in red and blue, respectively, and a merge image is also shown. Scale bars: 25 and 10 μm, respectively, for left and right panels. (B) UAP56 and DRM2 subcellular localization in flowers assessed by biochemical fractionation. Western blot analysis of total (T), cytosolic (C), and nuclear (N) protein extracts from WT plants. The cytosolic UGPase and nuclear histone H3 proteins are used as fraction cross‐contamination controls.

To determine in which cell compartment the aforementioned association occurs, we investigated UAP56 subcellular localization *in planta* through two independent approaches. First, we used transient co‐expression of *p35S‐DRM2‐RFP* and *p35S‐UAP56‐GFP* constructs in *N. benthamiana* leaves. DRM2–RFP chimeric protein triggers a diffuse nucleoplasmic signal with a distinct body excluded from nucleolus (mainly one body/nucleus) (Fig. 3A). The UAP56–GFP signal was partitioned between the nuclear and cytoplasmic compartments. In nucleus, UAP56–GFP protein was strictly detected in nucleoplasm, displaying a diffuse signal with some enriched zones organized rather as speckles. These transient assays indicated that both proteins co‐localized in nucleoplasm. A second approach achieved in Arabidopsis relied on protein immunodetection performed on subcellular fractions. We separated and enriched nuclear proteins from the ‘cytosolic’ fraction corresponding in fact to the remaining whole‐cell proteins. The efficiency of our nuclei/‘cytosol’ purification is illustrated in Fig. S4A, as the non‐phosphorylated state of the large RNA Pol II subunit is the main form detected in ‘cytosol’, whereas the total protein fraction presents also a slower migrating band corresponding to the elongation‐competent phosphorylated NRPB1 state 42. Considering the antibodies available in our study, this procedure prevents any bias that may come from promoter selection or protein tag addition. Although more global, the use of appropriate controls ensures high confidence. Thus, while DRM2 exhibited a nuclear exclusive pattern, the detection of UAP56 protein performed on these purified subcellular fractions supported a nucleocytosolic partitioning in flower cells (Fig. 3B), a result confirmed using distinct home‐made (Fig. S3) and previously tested antibodies 41. Nuclear histone H3 and cytosolic UGPase were used here as cross‐contamination controls. These latter observations were in agreement with all our transient assays conclusions. Previous studies detected UAP56 exclusively in the nucleus mainly in roots and leaves 41, 43. This difference can be easily explained by the nature of the organ and experiment used, suggesting an additional and yet unassigned function to UAP56 in reproductive organs in Arabidopsis.

### UAP56 nuclear fraction presents typical chromatin‐associated protein hallmarks in flowers

**Figure 4:**
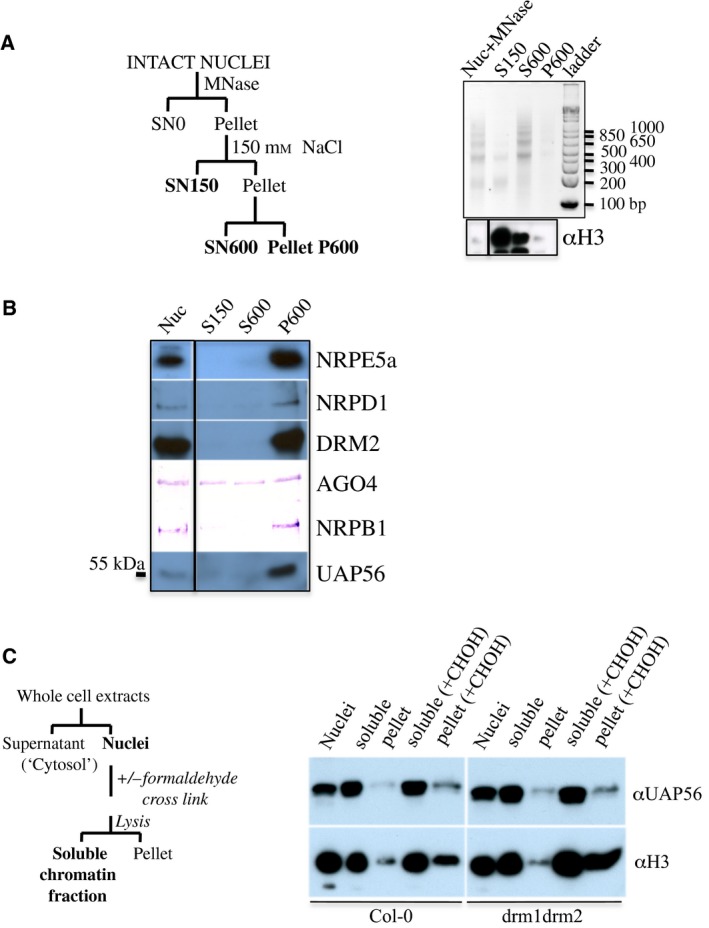
UAP56 is associated with chromatin. (A,B) Most of the UAP56 resides in salt‐resistant native chromatin fraction. (A) *In nucleo* digestion with MNase generates differential chromatin fractions depending on nucleosome accessibility, selectively separated with successive salts washes as described in scheme. Efficiency of digestion and fractionation of DNA purified from each native chromatin fractions is visualized by Gelred staining after electrophoresis on 2% agarose gel. In parallel, same fractions were controlled by western blotting, showing that histone H3 proteins are mainly retrieved in soluble fractions (bottom). (B) Immunodetection of UAP56, some transcription machinery such as RNA Pol II (NRPB1), Pol IV (NRPD1), Pol V (NRPE5a), and other RdDM core components (DRM2 and AGO4) in native chromatin fractions. (C) Procedure followed to perform chromatin extraction under denaturing conditions is schematized on left. The same suspension of nuclei in a detergent‐containing buffer (1% SDS) is submitted or not to a formaldehyde cross‐linking (±). A mechanical lysis and a centrifugation step allow separation of soluble and insoluble fractions. Profile of UAP56 is then analysed in Col‐0 and *drm1drm2* backgrounds by immunodetection presented on right. Anti‐H3 is used as nuclear control.

To further characterize the UAP56–DRM2 association in nucleus, we tested if UAP56 was indeed associated with chromatin in flowers and investigated further nuclear UAP56 signatures. Native chromatin digested by MNase can be separated through successive and increasing salt washes (Fig. 4A), allowing fractionation depending on nucleosome accessibility, the nature of protein association with chromatin and the solubility features of protein complexes 32. Low‐density nucleosome regions, such as enhancer or active regions, are more easily released, and condensed regions such as heterochromatin or large insoluble protein complexes are preferentially enriched in high salt resistant fractions (Fig. 4A). AGO4 protein, known to associate transiently with chromatin, was detected in all fractions (Fig. 4B). A chemical cross‐link prior to MNase action further stabilized AGO4 association to chromatin, facilitating its detection in the final pellet fraction. This observation provided an additional quality control to this experiment (Fig. S4B). Minor fractions of UAP56 and Pol II were retrieved in low salt‐extracted chromatin, but three RNA polymerases (Pol II, Pol IV, Pol V), DRM2 and UAP56 were mainly isolated in a final pellet step during native chromatin fractionation (Fig. S3B and Fig. 4B).

Next, we addressed the question of the impact of RdDM activity on UAP56 association to chromatin. To do this, we extracted chromatin from wild‐type or *drm1drm2* flowers, following standard preparation used for ChIP experiments (Fig. 4C). As this procedure is denaturant, a chemical cross‐link has been also applied in parallel before the mechanical DNA fragmentation to stabilize labile complexes and RNA‐mediated chromatin protein associations. This experiment confirmed that UAP56 is indeed mainly found in the enriched chromatin soluble fraction as DRM2 (data not shown for DRM2). Neither cross‐link treatment nor depletion of DRM2 modified UAP56 balance between the soluble and insoluble chromatin fractions. Altogether, these data support that UAP56 is tightly associated with chromatin and that its association is not strictly dependent on the presence of DRM2. This observation is not surprising as the expected contribution of the DRM2‐related function in UAP56's range of action is surely minor with respect to its splicing and mRNA exporting roles.

### As in yeast and animal counterparts, UAP56 activities may be crucial for plant development

In Arabidopsis, two tandemly duplicated genes (AT5G11170 and AT5G11200) have been shown to encode for 100% identical UAP56 proteins. This family also presents another specificity as knocking out one gene triggers compensation through the expression of the second gene to maintain equal level of transcript and protein 41. As an RNAi approach to knockdown of *UAP56* expression turned out to be unsuccessful for Kammel *et al*. 41, we carried out several strategies in parallel to obtain *uap56a/uap56b* double mutants to investigate a functional link with DRM2. First, we crossed the homozygous mutants *uap56a* and *uap56b* to generate F1 individuals that were hemizygous for each allele and analysed the F2 and F3 offspring for the presence of a line homozygous for both the *uap56a* and *uap56b* alleles, which we failed to identify. To complete this conclusion, we did not manage to isolate in these populations a plant exhibiting the double mutation in one gene and a single mutation in the second gene. We then outcrossed the double heterozygous F1 plant as pollen donor with a wild‐type plant, looking for plants that would present both *uap56* alleles as a consequence of a meiotic recombination event occurring between the two genes. However, despite the screening of a large F1 population (1462 plants), we failed to identify such an event. We finally tried the CRISPR/CAS9 gene‐editing method performed on each *uap56* single mutant. A sgRNA was designed to target the first exon of both genes 28. We found only 1 out of 60 plants analysed that displayed a mutation at the predicted site. This T1 plant with *uap56a/uap56a UAP56b/uap56b* genotype exhibited several development defects, affecting vegetative to adult transition (strong delay in development and reduced number of leaves), and reproduction (short and less siliques). The siliques contained also aborted ovules suggesting a defect in the fecondation process, leading to a reduced production of seeds. Unfortunately, we were unable to retrieve plants presenting the same genotype in the progeny. Finally, our unsuccessful attempts together with previously published data converge on the assumption of an essential role of UAP56 proteins in plant development.

## Conclusion

Several studies have already highlighted intricate relations between the RNA Pol II and RNA Pol IV/V machinery in transcription gene silencing 17, 20, 44. Here, we report that DRM2 and UAP56 are two interacting factors with affinity to chromatin. The underlying mechanism linking these two proteins and its implication for RdDM remain unclear, as we were unable to identify plants devoid of UAP56 activity. However, several observations suggest a possible functional convergence between these proteins. Indeed, both DRM2 and UAP56 proteins exhibit dsDNA binding activity *in vitro*, dsDNA being the DRM2 favored substrate in DNA methylation assays 20. DsDNA stimulates UAP56 ATPase activity, and uncouples its helicase and ATPase activities 41. All these properties can be expected for mechanisms involved in control of DNA methylation. In addition, our result regarding the intracellular partitioning of UAP56 in flowers may also be of a particular interest as UAP56 nucleocytosolic shuttling activity has also been shown to be functionally relevant in yeast and animal models. This activity is required in Drosophila for proper cytosolic localization of some specific transcripts impacting embryo axis specification 45, 46. All these data may argue in favor of the acquisition of additional and specific functions depending on organ or developmental stage, beyond splicing and mRNA export regulation. An attractive hypothesis is that UAP56 plays a new role through its partnership with DRM2 potentially linked to the RdDM pathway in reproductive organs. Knowing the functional links between UAP56 and Pol II, the most obvious hypothesis would be that UAP56/DRM2 association may contribute to the non‐canonical RdDM pathways initiated by Pol II transcription. Pol II feeds these alternative pathways with transcripts used for siRNA production through the action of diverse factors often shared with post‐transcriptional gene silencing machinery 25, 47, 48. Although this initiation phase differs from the classical RdDM pathway, the downstream chromatin‐bound effector phase requires Pol V and DRM2. As Pol II interacts with AGO4 24, Pol II transcripts can be potentially used as scaffolds to recruit the AGO4–siRNA complex in the vicinity of some RdDM target loci. In this context, two contributions of UAP56 can be assumed. First, UAP56 may simply stabilize DRM2 close to the Pol II–AGO4–siRNA complex in a chromatin environment that may differ from what can be observed for the classical RdDM pathway. A second contribution to consider involves UAP56 in a Pol II transcript sorting mechanism. Indeed, Pol II ensures the production of various classes of long non‐coding RNAs (lncRNAs), such as pri‐miRNA, *TAS* and endogenous repeat transcripts and these lncRNAs harbor the same Pol II‐specific signatures, such as a 5′‐cap and polyA tail. However, their fates are significantly different 49, some being dedicated to act in *cis* on RdDM targets, and others to be exported from the nucleus. The UAP56–DRM2 association may be used then as a sensor to discriminate between these populations of lncRNAs produced by Pol II. Indeed, the UAP56–DRM2 association may create a local excess of UAP56 concentration sufficient to block RNA export, a phenomenon that has previously been described in animal cells 38. This nuclear retention mechanism would thereby favor the Pol II transcript's fate to silence in *cis*. In this regard, the local dosage of UAP56 may also help to discriminate between genes and non‐genes transcribed by Pol II. In such a scenario, dynamic aspects such as intranuclear compartmentalization and the chromatin environment may also be determinant for the establishment of this sorting.

The impact of Pol II‐dependent non‐canonical pathways seems to be limited in wild‐type plants. These processes are characterized by cell specificity, a reduced number of targets known so far, and a transient action since they are also predicted to initiate expression‐dependent silencing before the targets switch into canonical RdDM. In this context, a genome‐wide mapping and quantification of Pol II, UAP56 and DRM2 through ChIP‐seq analyses may help to address fully the relevance of the DRM2–UAP56 partnership *in vivo*, and assess even a minor contribution of UAP56 to RdDM pathways or, more globally, to DRM2 action.

## Conflict of interest

The authors declare no conflict of interest.

## Author contributions

JA and TL conceived the study. Design of the experiments: JA and MAH. Performance of the experiments: JA, CP, LD and DP. Analysis of data: JA, CP and LD. MAH and SJK have contributed respectively to biochemical and MS/MS analyses. JA wrote the manuscript. All authors approved the manuscript.

## Supporting information


**Fig. S1.** Tools generated for DRM2 purification.

**Fig. S2.** Detailed procedure for DRM2 purification, followed by western blotting during the conventional chromatography separation.

**Fig. S3.** Production of anti‐UAP56 and anti‐NRPD1 specific antibodies.

**Fig. S4.** Controls for nuclear studies.

**Table S1.** List of all proteins identified by MS/MS common to both DRM2 purification strategies.

**Table S2.** List of the primers used in this study.
