# Supplementary figures and images for: UAP56 associates with DRM2 and is localized to chromatin in Arabidopsis
Source: FEBS Open Bio. 2019 Apr 5;9(5):973–85. doi: 10.1002/2211-5463.12627 (PMC6487834; doi:10.1002/2211-5463.12627)

FigS1

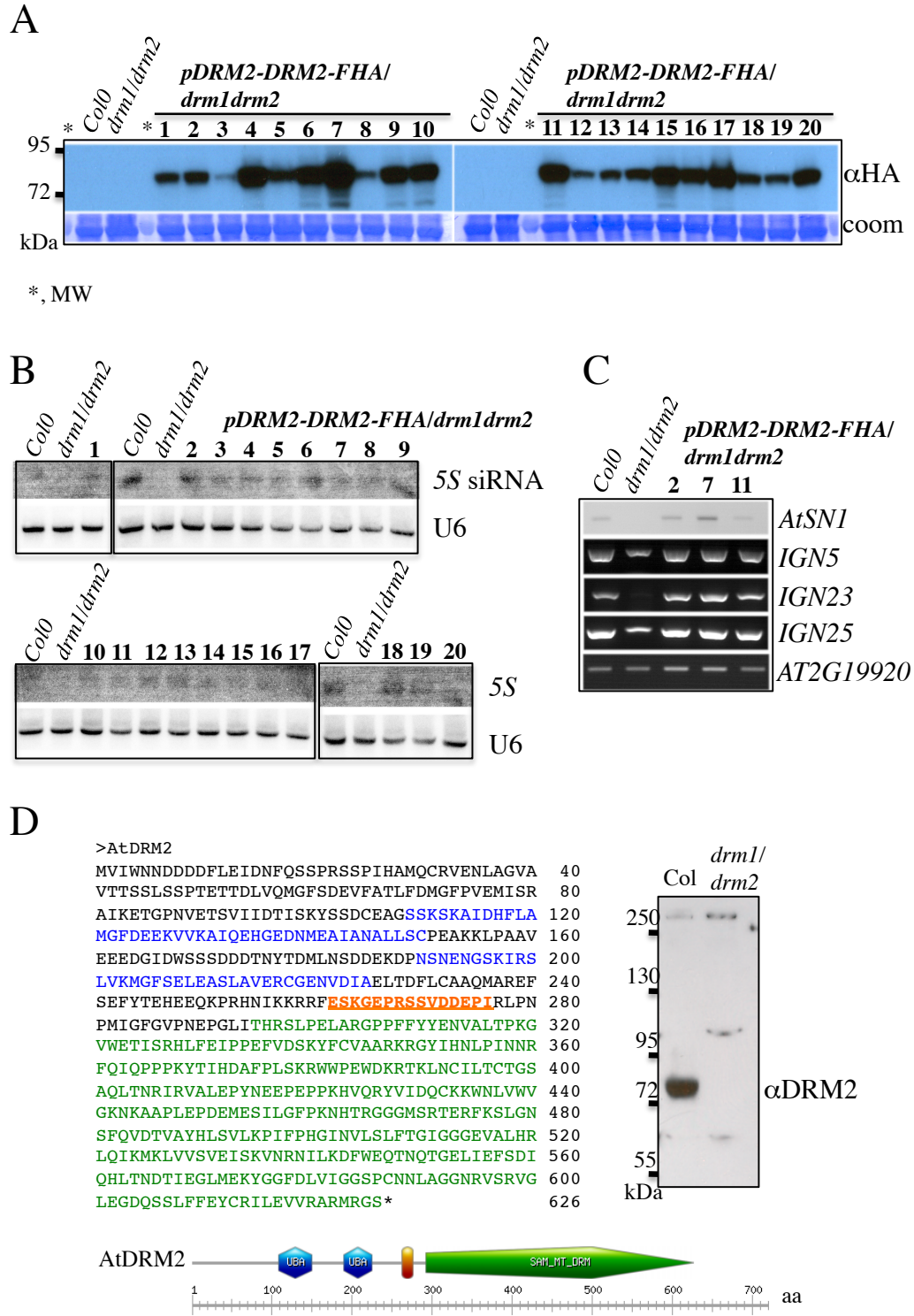

Supplement: Supplementary file 1 — Fig. S1. Tools generated for DRM2 purification. [file FEB4-9-973-s001.pdf]

FigS2

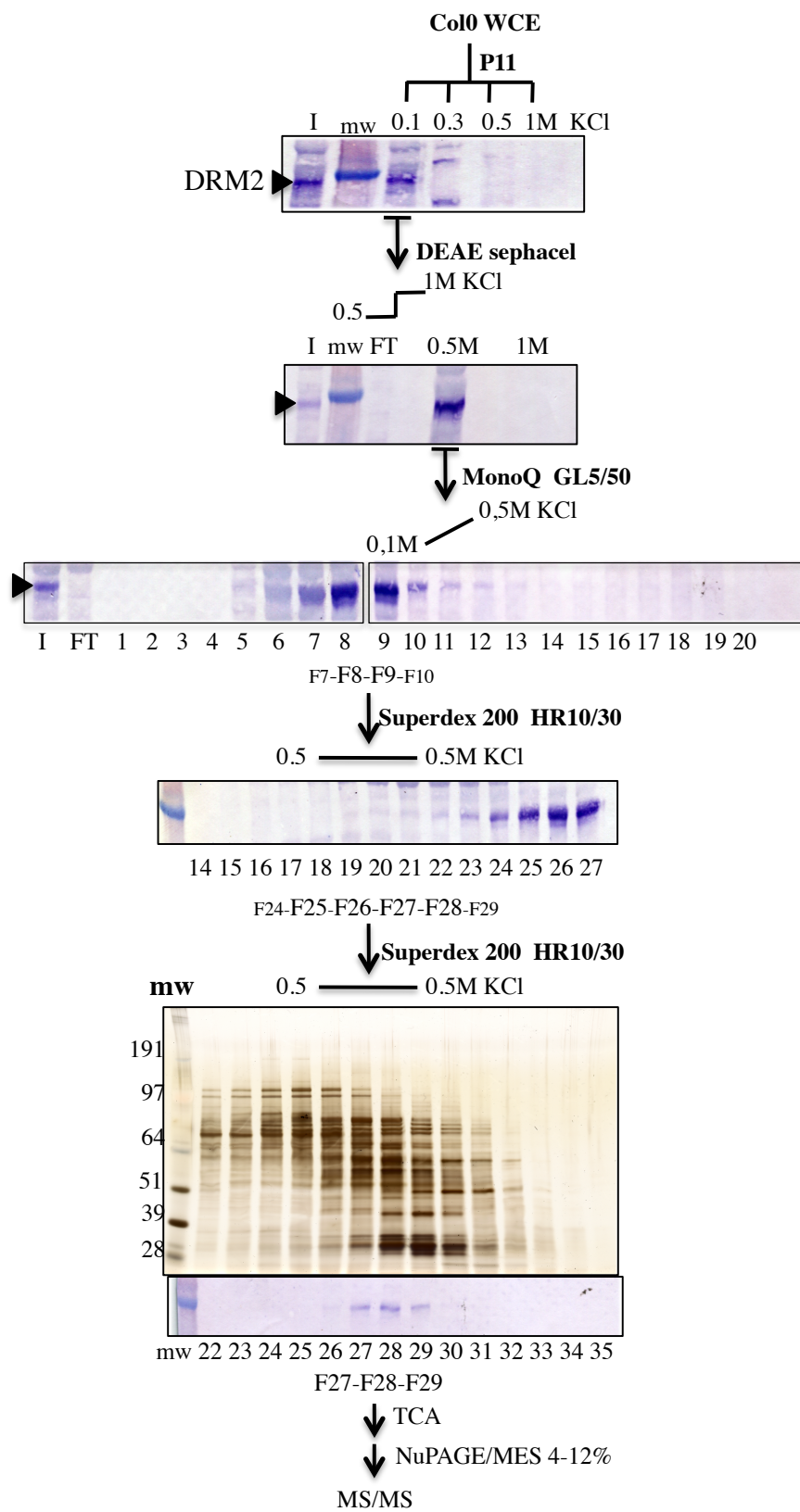

Supplement: Supplementary file 2 — Fig. S2. Detailed procedure for DRM2 purification, followed by western blotting during the conventional chromatography separation. [file FEB4-9-973-s002.pdf]

FigS3

A

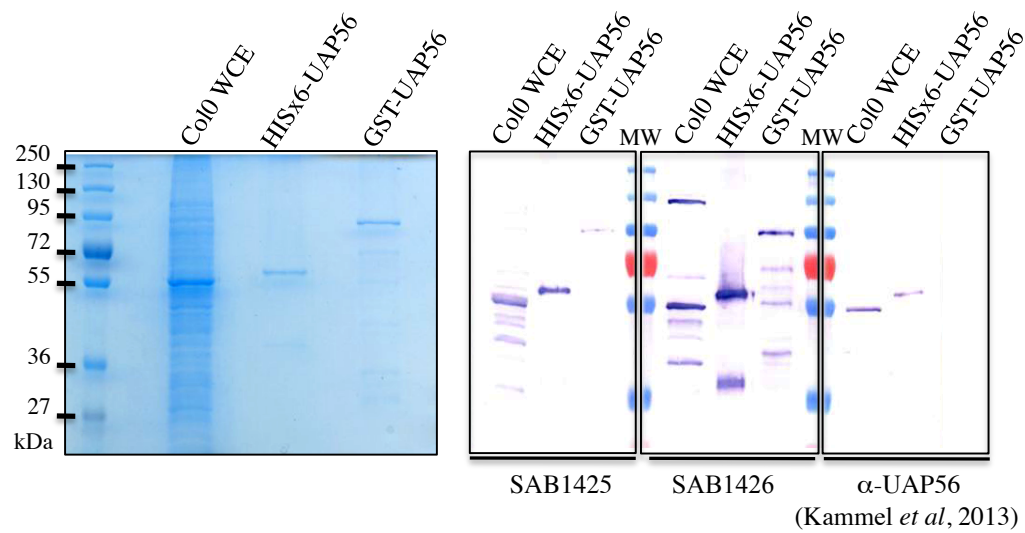

B

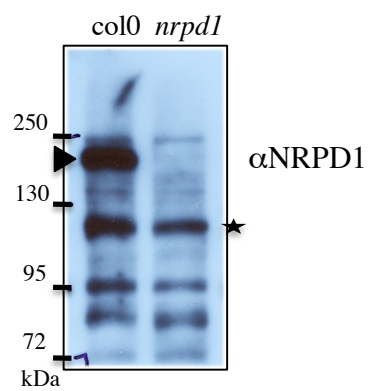

Supplement: Supplementary file 3 — Fig. S3. Production of anti‐UAP56 and anti‐NRPD1 specific antibodies. [file FEB4-9-973-s003.pdf]

FigS4

A

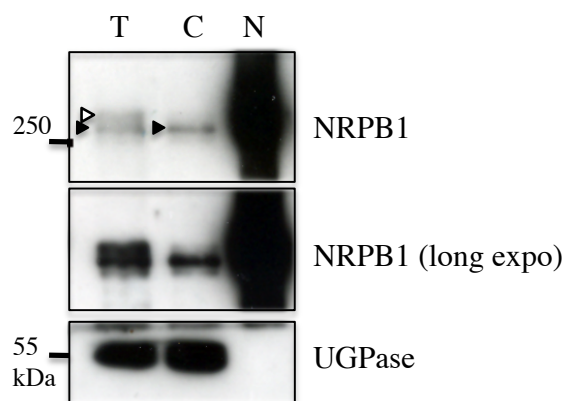

B

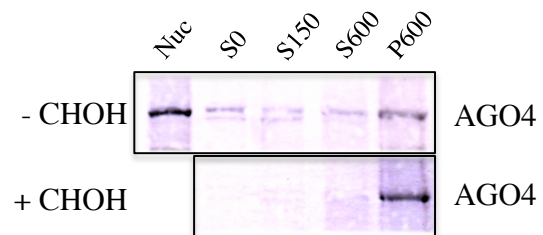

Supplement: Supplementary file 4 — Fig. S4. Controls for nuclear studies. [file FEB4-9-973-s004.pdf]
