# Supplementary material for: UAP56 associates with DRM2 and is localized to chromatin in Arabidopsis
Source: FEBS Open Bio. 2019 Apr 5;9(5):973–85. doi: 10.1002/2211-5463.12627 (PMC6487834; doi:10.1002/2211-5463.12627)
Supplement: Supplementary file 5 — Table S1. List of all proteins identified by MS/MS common to both DRM2 purification strategies. [file FEB4-9-973-s005.pdf]

Table S1

Proteins identified by MS/MS common to both DRM2 purification strategies

description	accession	pI	score	mass	coverage	#peptides	#specific peptides	emPAI
DRM2	AT5G14620.1	5,12	978,46	70385,92	42,81	21	21	2,69
HD domain-containing metal-dependent phosphohydrolase protein	AT5G40270.1	6,15	451,35	54638,67	25,16	10	10	0,85
HOG1	AT4G13940.1	5,66	347,70	53344,09	15,26	8	8	0,58
MSI2	AT2G16780.1	4,66	82,75	46674,64	5,78	2	2	0,14
ATTIP49A, RIN1	AT5G22330.1	5,67	58,50	50291,56	5,02	2	2	0,13
DEAD/DEAH box RNA helicase family protein	AT5G11170.1	5,42	131,49	48306,51	9,13	4	4	0,29
