# Supplementary material for: UAP56 associates with DRM2 and is localized to chromatin in Arabidopsis
Source: FEBS Open Bio. 2019 Apr 5;9(5):973–85. doi: 10.1002/2211-5463.12627 (PMC6487834; doi:10.1002/2211-5463.12627)
Supplement: Supplementary file 6 — Table S2. List of the primers used in this study. [file FEB4-9-973-s006.pdf]

Table S2

List of the primers used in this study

	**Sequence 5'-3'**
genomic *DRM2* cloning (XmaI)	CCCGGGGTGTGTGTGTATGTACACATGC
genomic *DRM2* cloning (BamHI)	GGATCCGATCCTCTCATCCTCGCACGTAC
DRM2 coding sequence cloning (SalI)	GTCGACATGGTGATTTGGAATAACGATG
DRM2 coding sequence cloning (PstI)	CTGCAGAGATCCTCTCATCCTCGCACG
UAP56 coding sequence cloning (SalI)	GTCGACATGGGAGACGCTAGAGACAAC
UAP56 coding sequence cloning (PstI)	CTGCAGAGAAGGCATGTAGGTTGAAGTAT C
genotyping *uap56b* SAIL_883C11 LP	CCCCTGCGGTATAAGACG
genotyping *uap56b* SAIL_883C11 RP	CTTTGCTTGGCAGATGACAT
genotyping *uap56a* GABI_528B02 LP	AACCAACTCTGCTCCTTATCTCAG
genotyping *uap56a* GABI_528B02 RP	GCTTGAGGGATACATTCA
5S siRNA probe	ATGCCAAGTTTGGCCTCACGGTCT
U6 probe	CTCGATTTATGCGTGTCATCCTTGC
Chop-PCR for IGN5, Fwd	TCCCGAGAAGAGTAGAACAAATGCTAAA A
Chop-PCR for IGN5, Rev	CTGAGGTATTCCATAGCCCCTGATCC
Chop-PCR for IGN23, Fwd	ACTGAAAATTGTAAACAAAGAAACGGCA CTACA
Chop-PCR for IGN23, Rev	GATCGGTCCATAAACTTGTTGGGTTT
Chop-PCR for IGN25, Fwd	CTTCTTATCGTGTTACATTGAGAACTCTTT CC
Chop-PCR for IGN25, Rev	ATTCGTGTGGGCTTGGCCTCTT
Chop-PCR for AT2G19920, Fwd	GGAGAGAGGCTTGTTGGATACTGC
Chop-PCR for AT2G19920, Rev	GAACACGCATGACAGTGGGTGGAG
UAP56 guide for CRISPR/CAS9, Fwd	GATTGGAGACAACGAAGCCTACG
UAP56 guide for CRISPR/CAS9, Rev	AAACCGTAGGCTTCGTTGTCTCC
PCR screening for CRISPR editing *UAP56a/b*, Fwd	TCGAGAACTCAATTGCTTATC
PCR screening for CRISPR editing in *UAP56a* specific, Rev	TAGATAATTGAAGAACTTAGTCGG
PCR screening for CRISPR editing in *UAP56b* specific, Rev	GCAACTAATTAAACACATACTGCTAC
