# Supplementary material for: UAP56 associates with DRM2 and is localized to chromatin in Arabidopsis
Source: FEBS Open Bio. 2019 Apr 5;9(5):973–85. doi: 10.1002/2211-5463.12627 (PMC6487834; doi:10.1002/2211-5463.12627)
Supplement: Supplementary file 7 [file FEB4-9-973-s007.docx]

SUPPORTING INFORMATION

**Figure S1.** Tools generated for DRM2 purification. A-C) Production of the complemented *DRM2* tagged lines. (A) Expression of DRM2-FLAG-HA protein in T1 plants analysed by western blotting using anti-HA antibodies; (B) complementation of the *5S* siRNA accumulation defect observed in *drm1drm2* mutant assayed by LMW Northern blots, U6 is used here as loading control; (C) complementation of DNA methylation defects on RdDM targets analysed by CHOP PCR. Genomic DNA from wild type, *drm1drm2* mutant, and three representating tagged lines exhibiting different protein accumulation level, are digested with the methylation sensitive restriction endonuclease HaeIII. The loci targeted are then amplified by PCR, and AT2G19920 is used as an undigested control. D) Production and test of the anti-DRM2 antibodies. The epitope selected to raise antibodies is excluded from AtDRM2 conserved domains and is depicted in red in DRM2 amino acid sequence and in a graphical view of the protein domains organisation (https://prosite.expasy.org/cgi-bin/prosite/mydomains). The serum produced in rabbit is affinity-purified and detects specifically DRM2 protein by western blotting on wild type Col0 compared to *drm1drm2* flowers extracts.

**Figure S2.** Detailed procedure for DRM2 purification, followed by western blotting during the conventional chromatography separation. Fractions used in each step, and DRM2 immunodetection (by colorimetry method) are indicated with arrows. Silver staining of fractions obtained from last gel filtration Superdex200 HR10/30. Fractions 27-28-29 were selected and pooled for TCA precipitation, separated by electrophoresis and analysed further by MS/MS. FT, Flowthrough; MW, protein ladder.

**Figure S3.** Production of anti-UAP56 and anti-NRPD1 specific antibodies. A) UAP56 antibodies produced against entire protein are tested by western blot on *A. thaliana* whole cell extracts and on two purified recombinant proteins (Hisx6 or GST tagged proteins). Immunodetection with indicated sera is performed using colorimetry method, and a colloidal blue staining corresponding to gel loading is shown. B) New production of anti-NRPD1 serum in rabbit (Eurogentec), using peptide LKNGTLESGGFSENP as antigen (previously designed in [8]). Affinity-purified anti-NRPD1 antibodies react specifically with NRPD1 in a wild type flowers whole cell extract compared to *nrpd1-4* mutant extracts (Salk_083051). NRPD1 corresponding band is shown with an arrow, and a parasite band indicated by an asterisk is used here as loading control.

**Figure S4.** Controls for nuclear studies**.** A) Cell fractionation purity obtained with the protocol described in methods was assayed using RNA Pol II CTD repeats signatures as this domain becomes specifically phosphorylated when engaged in a transcription elongation-competent complex. Consequently, the slower migrating Pol II phosphorylated form (empty arrow) observed in total fraction is indeed excluded from cytosol as depicted in this blot. The non-phosphorylated Pol II large subunit is indicated by a black arrow. B) Formaldehyde cross-linking stabilizes transient interactions to chromatin. To illustrate this, the AGO4 distribution into salt extracted native chromatin fractions is visualized by immunodetection (colorimetry). Chemical cross-linking performed or not (+/- CHOH) on the same nuclei suspension prior to MNase digestion triggers a shift of AGO4 from low salt fractions to the high salt fraction enriched in core components of RdDM machineries. S0, S150, S600 correspond to soluble fractions obtained after centrifugation on nuclei suspension MNase digestion directly (S0, 0 mM NaCl), or after NaCl washes (S150, 150 mM; S600, 600 mM), and final resistant salt pellet (P600, 600 mM).
